# Variability and associated factors in the management of cord clamping and the milking practice among Spanish obstetric professionals

**DOI:** 10.1038/s41598-020-58641-z

**Published:** 2020-02-03

**Authors:** Inmaculada Ortiz-Esquinas, Juan Gómez-Salgado, Ana Isabel Pascual-Pedreño, Julián Rodríguez-Almagro, Ana Ballesta-Castillejos, Antonio Hernández-Martínez

**Affiliations:** 1 Department of Obstetrics & Gynaecology, Alcázar de San Juan, Ciudad Real, 13600 Spain; 2 Department of Sociology, Social Work and Public Health, University of Huelva, 21071 Huelva, Spain; 3 Safety and Health Postgraduate Programme, Espíritu Santo University, Guayaquil, 091650 Ecuador; 4 Department of Nursing, Ciudad Real School of Nursing, University of Castilla La-Mancha, Ciudad Real, 13071 Spain; 5 Department of Obstetrics & Gynaecology, Talavera de la Reina Hospital, Toledo, 45600 Spain

**Keywords:** Health policy, Quality of life, Risk factors

## Abstract

Clinical practice guides recommend delayed clamping of the umbilical cord. If this is not possible, some authors suggest milking as an alternative. The objective of this study was to determine the variability in professional practice in the management of umbilical cord clamping and milking and to identify factors or circumstances associated with the different methods. An observational cross-sectional study done on 1,045 obstetrics professionals in Spain in 2018. A self-designed questionnaire was administered online. The main variables studied were type of clamping and use of milking. Crude odds ratios (OR) and adjusted odds ratios (ORa) were estimated using binary logistic regression. 92.2% (964) performed delayed clamping. 69.3% (724) clamped the cord when it stopped beating. 83.8% (876) had heard of milking, and 55.9% (584) had never performed it. Professionals over 50 were less likely to perform delayed clamping, with an ORa of 0.24 (95% CI: 0.11–0.52), while midwives were more likely to perform delayed clamping than obstetricians, with an ORa of 14.05 (95% CI: 8.41–23.49). There is clinical variability in the management of umbilical cord clamping and the use of milking in normal births. Part of this variability can be attributed to professional and work environment factors.

## Introduction

Umbilical cord clamping and cutting are done in the third stage of labour. Two types of clamping have been described, depending on how long after birth they are performed: early and delayed. In early umbilical cord clamping (ECC), the cord is clamped within the first minute after birth, while in delayed cord clamping (DCC), the clamping is done between one and three minutes of birth, or when the cord stops beating^1–4^. It should be pointed out that, since 2010, ILCOR recommend that the cord should not be clamped within one minute^5^.

Among the advantages for the newborn, it has been observed that DCC in term newborns resulted in increased levels of haemoglobin at birth and, consequently, better iron levels in the first few months of life, which can have a favourable impact on the child’s development^6–9^. In the case of preterm newborns, a reduction in rates of intraventricular haemorrhage and necrotising enterocolitis has been observed, as well as a reduced need for transfusion. If ECC is performed, there is an increase in the mortality rate with respect to DCC^10^. However, an increased risk of jaundice has been described^11^. With regard to mothers, in 2013, McDonald *et al*. concluded that DCC was not related to an increased risk of postpartum haemorrhage (PPH) or a difference in postpartum haemoglobin levels compared with early clamping^2^.

Due to the impact that clamping has on maternal-foetal health^3,6,8–11^ international Clinical Practice Guides (CPGs) recommend this procedure DCC in all births, when possible^12–15^.

When DCC is not possible for any reason, such as immediate neonatal resuscitation or maternal haemodynamic instability, umbilical cord milking (UCM)has been proposed as an alternative option to DCC. This technique consists of milking the umbilical cord several times along 10 or 20 cm of the cord’s length, from the placenta toward the newborn^16,17^. While cord milking has risks in extremely preterm infants^18^ it has been shown to be safe in term and near term infants^19,20^.

Wu *et al*. found that umbilical cord drainage decreased the duration of the third stage of labour by 2.28 minute, but the amount of blood loss was not reduced. In case of normal vaginal deliveries, the incidence of postpartum haemorrhage was decreased by 3%^21^.

Despite the fact that benefits for the newborn have been demonstrated, more studies are needed in order to recommend this practice^13,22,23^.

Studies on current practices among health professionals in different countries have shown that the majority of professionals perform DCC, existing variability with regard to when it is performed^24–27^.

Currently, in Spain, although there is a concern about pregnant women’s health^28,29^,there is an overall lack of information on umbilical cord clamping types and techniques used by professionals, as well as on the frequency at which UCM is performed and the reasons for using this procedure. For this reason, and due to the significant implications that cord clamping has for newborns, our objective was to determine the variability in clinical practice in the management of umbilical cord clamping and to identify the professional or work environment factors associated with the different ways of managing it.

## Methodology

### Design and selection of study subjects

An observational cross-sectional study was conducted involving obstetrics professionals (obstetricians, midwives, and students) in Spain during 2018^30^.

### Study population and sample

The study population were obstetrics professionals including obstetricians, obstetrician students, midwives, and midwifery students. Primary care obstetrics professionals were excluded as they do not attend childbirths.

According to official statistics offered by the Spanish Ministry of Health, Consumer Affairs and Social Welfare, a population of 16,361 obstetrics professionals were registered in Spain. Out of them, 9,013 were midwives, 743 were midwifery students, 5,616 were obstetricians, and 989 obstetrics students^31,32^.

To estimate the sample size, the following criteria were considered. As it was a multiple-choice questionnaire in which the prevalence of each response option was unknown, a prevalence of 50% was used for being the criterion that requires the largest sample size. Additionally, a confidence level of 95% was stated and a precision or absolute error of 3%, giving a minimum sample size of 1,002 study subjects. For this estimation, the EPIDAT 4.1 software was used^30^.

### Data collection

To collect the information, a self-designed online anonymous questionnaire was used containing 35 items on sociodemographic data, professional and work environment characteristics, and different ways of performing cord clamping and UCM.

The questionnaire was distributed to obstetrics professionals via the Spanish Midwives Associations Federation and its sixteen member associations (https://www.federacion-matronas.org/). The questionnaire was also distributed through Scientific Societies of Obstetrics^30^.

Once the study subjects agreed to participate, they were given instructions on completing the questionnaire. An email address was set up to respond to questions or issues raised in relation to filling out the questionnaire.

The following variables were collected:

The main dependent variables were type of clamping (ECC or DCC) and use of UCM (No/Yes). Other dependent variables were waiting time before clamping the umbilical cord in DCC (less than 1 minute/1–2 minutes/more than 2 minutes/when the cord stops beating).

The independent variables were: age, sex, profession (midwife/midwifery student/ obstetrician/obstetricsstudent), hospital(public or private), works in a private hospital (No/Yes), home births (No/Yes), number of birth per year at the hospital (≤1000 births/1001–2000 births/2001–4000 births/>4000 births), existence of students at the hospital (no students/onlymidwifery students/only obstetricsstudents/both specialties’ students) and year of completion of training (before 2007/between 2007 and 2013/after 2013/in training).

### Statistical analysis

First, a descriptive analysis was done using absolute and relative frequencies. For the questions related with umbilical cord clamping and UCM, a weighting factor analysis was used for the variable ‘profession’, with the aim of improving the representativeness of the sample. The weighting factor was obtained by dividing the relative frequency of the theoretical sample by the relative frequency of the real sample obtained in our study. Weighting is a statistical technique that can be used to correct any imbalance in sample profiles after data collection. The weighting coefficients were 0.73 (0.55/0.75) for midwives, 0.49 (0.04/0.09) for midwifery students,3.12 (0.34/0.11) for obstetricians, and 1.44 (0.06/0.04) for obstetrics students^30^.

Next, a bivariate analysis of the different sociodemographic and professional factors in relation to the type of clamping was done using binary logistic regression. Then, a multivariate analysis was performed through binary logistic regression using SPSS forward and backward selection, and potential confounders were included in the analysis. The crude (OR) and adjusted (ORa) odds ratios were estimated with a respective confidence interval of 95% (CI95%) related with the type of clamping and its relationship with the professional and work environment characteristics.

### Ethical approval

This study was approved by the Clinical Research Ethics Committee (CEIC) of Mancha-Centro Hospital with identification number: 91-C. Before starting the questionnaire, obstetrics professionals read a fact sheet about the study, its objectives, etc., and marked a box by which they showed their consent to participate in it, i.e., they signed an online informed consent (ticking the option if they wanted to participate or not doing so when refusing to take part in the study). we followed the protocols established to carry out this type of research with the purpose of publication/disclosure to the scientific community. The study was conducted according to the strobe guidelines set in the Declaration of Helsinki and all procedures involving human subjects were approved by the Ethics Committee. All women involved in this study filled out informed consent and data treatment forms to enter the study, in accordance with the ethical standards of the Ethics Committee.

## Results

### Professional and work environment characteristics

1,045 professionals participated in this study, which means a response rate of 8.7% midwives, 13.0% midwifery students, 2.0% obstetricians, and 4.4% obstetrics students.

Out of the study sample, 75.5% (n = 789) were midwives, 11.0% were obstetricians (n = 115), and 13.5% (n = 141) midwifery students or obstetrics students. 89.4% (n = 934) of the sample were women, 33.1% (n = 346) finished their specialist training after 2013, and 3.6% (n = 38) attended home births. Table 1 summarises the sociodemographic, professional and work environment characteristics.

**Table 1:** Sociodemographic, professional, and work environment characteristics.

Variable	% (n)
**Age**	
≤30	36.3 (379)
31–40	33.4 (349)
41–50	210 (20.1)
>50	10.2 (107)
**Sex**	
Male	10.6 (111)
Female	89.4 (934)
**Profession**	
Midwife	75.5 (789)
Midwifery student	9.3 (97)
Obstetrician	11.0 (115)
Obstetrics student	4.2 (44)
**Year of completion of training**	
Before 2007	26.7 (279)
2007–2013	26.6 (278)
After 2013	33.1 (346)
In training	13.6 (142)
**Works in a public hospital**	
No	3.5 (37)
Yes	96.5 (1008)
**Works in a private hospital**	
No	85.8 (897)
Yes	14.2 (148)
**Attends home births**	
No	96.4 (1007)
Yes	3.6 (38)
**Works in Primary Care**	
No	81.1(847)
Yes	18.9 (198)
**Number of births per year in their hospital**	
<1000 births	24.1 (252)
1001–2000 births	32.7 (342)
2000–4000 births	26.9 (281)
>4000 births	16.3 (170)
**Professionals in training at the hospital**	
No professionals in training	18.5 (193)
Midwifery students only	4.3 (45)
Obstetrics students only	5.4 (56)
Both specialities	71.9 (751)

### Clamping types and techniques

With regard to the type of clamping used by professionals, 92.2% (n = 964) performed DCC. When weighted for profession, the overall percentage of DCC fell to 84.2%. With respect to the time waited to clamp the cord in DCC, 69.3% (n = 724) clamped the cord when it stopped beating, while 14.1% (n = 147) waited 1–2 minutes to clamp it.

### Use of milking and milking techniques

83.8% (n = 876) of professionals knew what UCM was. 55.9% (n = 584) had never performed it, while 3.4% (n = 36) performed it frequently. 49.6% (n = 216) performed UCM when it was not possible to perform DCC. 17.1% (n = 79) only performed it in premature births and 7.8% (n = 36) performed it systematically. With regard to the number of times professionals milked the umbilical cord toward the newborn, 29.9% (n = 138) milked it once, while 25.6% (n = 118) didnot milk it a set number of times. On carrying out the analysis with the weighting factor for the variable ‘profession’, significant differences were observed with respect to the unweighted analysis in the questions related to the situations in which milking was used and the number of times the cord was milked. Table 2 shows the type of clamping, use of UCM, and the technical characteristics of the procedures.

**Table 2:** Type of clamping, use of milking, and technical characteristics.

Variable	% (n)	% (n) Weighted
**Type of umbilical cord clamping habitually used**		
Early	7.8 (81)	15.0 (158)
Delayed	92.2 (964)	84.2 (887)
**Time until umbilical cord is clamped if delayed clamping is performed**		
Less than 1 minute	2.5 (26)	2.4 (21)
1–2 minutes	14.1 (147)	15.9 (141)
More than 2 minutes	11.4 (119)	10.4 (93)
When the cord stops beating	69.3 (724)	70.9 (629)
Missing values	2.8 (29)	0.3 (3)
**Do you know what umbilical cord milking is?**		
No	16.2 (169)	14.8 (155)
Yes	83.8 (876)	85.2 (890)
**Have you ever performed cord milking?**		
Never	55.9 (584)	51.0 (534)
Rarely	23.4 (245)	23.5 (246)
Occasionally	16.5 (172)	20.4 (213)
Frequently	3.4 (36)	4.6 (48)
Always	0.8 (8)	0.5 (5)
**Situations that lead to use milking**		
Systematically in all births	7.8 (36)	3.2 (33)
Only in premature births	17.1 (79)	11.7 (122)
When delayed clamping cannot be used due to the need to perform neonatal resuscitation	49.6 (216)	22.7 (237)
I have more than a single criterion	28.2 (130)	62.5 (653)
**Number of times the cord is “milked” during milking**		
Once	29.9 (138)	14.7 (154)
Twice	21.7 (100)	11.1 (116)
Three times	19.5 (90)	10.1 (106)
Four times	2.2 (10)	1.1 (12)
Five times	1.1 (5)	1.0 (11)
I don’t have established criteria	25.6 (118)	61.9 (647)
**At your hospital, is there a protocol for the management of labour?**		
No	27.7 (289)	25.6 (267)
Yes, but each professional applies his/her own criteria	21.2 (222)	19.9 (208)
Yes, and the majority of professionals apply it	51.1 (534)	54.5 (570)

### Factors associated with the type of clamping

The professional and work environment characteristics associated with the use of DCC were analysed. When carrying out the multivariate analysis, it was observed that the older the health professional, the lower the probability of using DCC, with professionals over 50 having an OR of 0.24 (95% CI: 0.11–0.52), as compared with professionals aged 30 and under. On the other hand, it was observed that midwives had an OR of 14.05 (95% CI: 8.41–23.49) of performing DCC compared with obstetricians. Professionals from hospitals with between 1,001 and 2,000 births per year increased the probability of performing DCC, with an OR of 2.72 (95% CI: 1.35–5.47) compared with those with less than 1,000 births per year. The bivariate and multivariate analysis are shown in Table 3.

**Table 3:** Type of clamping and its relationship with professional and work environment characteristics.

Variable	Type of clamping		Bivariate analysis OR CI 95%	Multivariate analysis *ORa CI 95%
	Early (N = 81) % (n)	Delayed (N = 964) % (n)		
**Age**				
≤30	5.0 (19)	95.0 (360)	1 (ref.)	1 (ref.)
31–40	7.7 (27)	92.3 (322)	0.62 (0.34–1.15)	0.72 (0.37–1.40)
41–50	7.6 (16)	92.4 (194)	0.64 (0.32–1.27)	0.62 (0.29–1.33)
>50	17.8 (19)	82.2 (88)	**0.24 (0.12–0.48)**	**0.24 (0.11–0.52)**
**Sex**				
Male	13.5 (15)	86.5 (96)	1 (ref.)	
Female	7.1 (66)	92.9 (868)	**2.05 (1.12–3.74)**	
**Profession**				
Obstetrician	31.4 (50)	68.6 (109)	1 (ref.)	1 (ref.)
Midwife	3.5 (31)	96.5 (855)	**12.65 (7.74–20.66)**	**14.05 (8.41–23.49)**
**Completion of training**				
Before 2007	11.8 (33)	88.2 (246)	1 (ref.)	
Between 2017 and 2013	6.8 (19)	93.2 (259)	**1.82 (1.01–3.30)**	
After 2013	4.3 (15)	95.7 (331)	**2.96 (1.57–5.57)**	
Training period	9.4 (14)	90.1 (128)	1.22 (0.63–2.37)	
**Works in a public hospital**				
No	8.1 (3)	91.9 (34)	1 (ref.)	
Yes	7.7 (78)	92.3 (930)	1.05 (0.31–3.50)	
**Works in a private hospital**				
No	7.0 (63)	93.0 (834)	1 (ref.)	
Yes	12.2 (18)	87.8 (130)	**0.54 (0.31–0.95)**	
**Attends home births**				
No	8.0 (81)	92.0 (926)	NC	
Yes	0.0 (0)	100.0 (38)	NC	
**Number of births per year in their hospital**				
<1000 births	10.7 (27)	89.3 (225)	1 (ref.)	1 (ref.)
1001–2000 births	5.0 (17)	95.0 (325)	**2.29 (1.22–4.30)**	**2.72 (1.35–5.47)**
2000–4000 births	8.9 (25)	91.1 (256)	1.22 (0.69–2.17)	1.13 (0.59–2.14)
>4000 births	7.1 (12)	92.9 (158)	1.58 (0.77–3.21)	1.83 (0.82–4.06)
**Professionals in training at the hospital**				
No professionals in training	9.3 (18)	90.7 (175)	1 (ref.)	
Midwifery students only	2.2 (1)	97.8 (44)	4.52 (0.58–34.82)	
Obstetrics students only	14.3 (8)	85.7 (48)	0.61 (0.23–1.50)	
Both specialities	7.2 (54)	92.8 (697)	1.32 (0.75–2.32)	

In bold, the variables that presented a statistically significant relationship.

## Discussion

In the present study we observed that 92.2% of professionals performed DCC, with a great variability in the amount of time until the cord was clamped when delayed clamping was used. Also, DCC was associated with certain professional characteristics such as the age of the professional, the profession, and the number of births at the hospital.

Furthermore, 83.3% of professionals knew what UCM was, with significant variability in terms of situations in which UCM was carried out and the number of times the umbilical cord was milked by using this technique.

The physiological transition from foetal to neonatal circulation occurs through the redistribution of residual blood from the placenta to the newborn, which is known as placental transfusion. The physiological closure of the umbilical vessels ends with this placental transfusion. Clamping the cord after the physiological closure of the vessels improves adaptation to extrauterine life as it optimises the filling of the pulmonary vessels. When the UCC is performed, it improves pulmonary blood flow immediately at birth and helps lung expansion at the beginning of breaths^33,34^.

The WHO recommends^1^ performing umbilical cord clamping between 1–3 minutes after birth. In our study, around 80% of professionals follow this recommendation. This is a strong recommendation and adherence is a reasonable measure of good-quality care.

With regard to the prevalence of DCC, Boere *et al*.^25^ conducted a study of 500 professionals in the Netherlands. Results showed that90% used DCC in vaginal births without complications, with figures very similar to the ones shown in our study. Meanwhile, a study conducted by Leslie *et al*. in USA on a sample of 171 obstetricians found that 67% used DCC. Lundberg *et al*. conducted a study of 50 obstetrics departments in Norway that found that 76% used DCC^35^.

The 2014 NICE^12^ recommend do not clamp the cord earlier than 1 minute from the birth of the baby. Systematic reviews have revealed that DCC has beneficial short-term and long-term effects for the newborn. Among its benefits are: increased levels of haemoglobin, improved iron deposits, a reduction in rates of intraventricular haemorrhage and necrotising enterocolitis, as well as a reduced need for transfusion,reduce infant anemia, apart from not increasing the incidence of obstetric complications in mothers^2,36^.

The optimum time to clamp the umbilical cord has not been established yet^2,8,9,24^. In another study, 69.3% of professionals clamped the cord once it stopped beating. In this regard, and coinciding with our results, Boere *et al*.^25^ observed that 54% of professionals in the Netherlands that used DCC waited until the cord stopped beating to clamp it.Many authorities have pointed out that the precise time of clampingis not as important and the stage of physiological transition of the neonate^37^.

It is also possible to explain the increased use of DCC through professional factors. Specifically, we observed a greater use of DCC among midwives than among obstetricians, concurring with the work done by Farrar *et al*.^38^ and Boere *et al*.^25^.

Another factor related with an increased use of DCC in our study was the size of the hospital. In hospitals with between 1,001 and 2,000 births per year, there was a greater probability of DCC being used than in hospitals with less than 1,000 births. This difference was not observed with respect to larger hospitals, despite the fact that, on average, larger hospitals tend to have better quality indicators^39^.

Dalheim *et al*.^40^ identified several factors as barriers to the application of evidence-based clinical practice, one of which is age. In our study, we identified that one of the factors associated with a lower probability of using DCC was age. Professionals aged over 50 used delayed clamping less than professionals under 30.

Furthermore, when it is not possible to perform DCC, some professionals opted to perform milking. In our study, 17.1% of professionals performed UCM when the baby was preterm. In the US^25^, the prevalence of the use of UCM in newborns was 38.6%, while in the study done in 50 obstetrics departments in Norway, 6 of them reported that they used UCM in babies born before 32 weeks’ gestation^41^.

According to an Italian study, in the case of term newborns via caesarean, if delayed clamping was not possible, UCM could be considered as an alternative procedure to increase haemoglobin levels in the postnatal period and iron reserves in the following weeks^42^. The Royal College of Obstetricians and Gynaecologist^39^ also considers it as an alternative to delayed clamping, but more in-depth research is required to evaluate the differences between preterm newbornsandterm newborns, as well as the associated benefits and risks, before it can be implemented systematically.

In our study, half of the professionals that used UCM did so in situations where DCC could not be used, with wide variability in the number of times the cord was milked toward the newborn. No exact number of times to milk the cord has been established, although several studies establish this number at between two and four^41,43–45^.

One limitation of the study is the possibility of a selection bias in its design due to the fact that more midwives than obstetricians participated. However, this is a reflection of actual practice in Spain, as normal eutocic births are usually attended by midwives. In this regard, we carried out an additional analysis using profession as a weighting factor. One of the biggest strengths of this study is that it is the first one conducted in Spain to find out how umbilical cord clamping is performed, with a large sample which reveals the variability among professionals. Furthermore, the results of this study can serve as a basis for new research in this field to establish comparisons and healthcare policies aimed at improving professional training.

## Conclusions

There is significant variability among obstetrics professionals with respect to the type of clamping and milking of the umbilical cord regarding normal births. Most professionals perform DCC, presenting a great variability in the waiting time to clamp the umbilical cord.

The DCC was associated with certain professional characteristics such as the age of the professional, the profession, and the number of births at the workplace. Thus, being of lower age, a midwife and working in a centre with 1,001–2,000 births per year increased the likelihood of performing DCC.

On the other hand, most professionals know about the milking technique and use it as an alternative when they cannot perform DCC.

More research is needed to determine the most appropriate procedures, which can then serve as the basis for professionals to draw up consensus statements and reduce the variability in clinical practice.

## Data Availability

The data sets generated and/or analysed during the current study are available from the corresponding author on reasonable request.

## References

[1] *II WHO I Guideline: Delayed umbilical cord clamping for improved maternal and infant health and nutrition outcomes*. 26269880

[2] McDonald, S. J., Middleton, P., Dowswell, T. & Morris, P. S. Effect of timing of umbilical cord clamping of term infants on maternal and neonatal outcomes. *Cochrane Database Syst. Rev*. CD004074. 10.1002/14651858.CD004074.pub3 (2013). 10.1002/14651858.CD004074.pub3 PMC6544813 23843134

[3] *Grupo de trabajo de la Guía de Práctica Clínica sobre Atención al Parto Normal. Guía de Práctica Clínica sobre la Atención al Parto Normal. Plan de Calidad para el Sistema Nacional de Salud del Ministerio de Sanidad y Política Social. Agencia de Evaluació*.

[4] World Health Organization. *Recom. la OMS para la prevencin y el Trat. la hemorragia posparto Internet Available from* wwwwhoint (2014).

[5] Perlman J. M., Wyllie J., Kattwinkel J., Atkins D. L., Chameides L., Goldsmith J. P., Guinsburg R., Hazinski M. F., Morley C., Richmond S., Simon W. M., Singhal N., Szyld E., Tamura M., Velaphi S. (2010). Part 11: Neonatal Resuscitation: 2010 International Consensus on Cardiopulmonary Resuscitation and Emergency Cardiovascular Care Science With Treatment Recommendations. Circulation.

[6] Westhoff, G., Cotter, A. M. & Tolosa, J. E. Prophylactic oxytocin for the third stage of labour to prevent postpartum haemorrhage. *Cochrane database Syst. Rev*. CD001808, 10.1002/14651858.CD001808.pub2 (2013). 10.1002/14651858.CD001808.pub2 24173606

[7] Dunn PM (2003). Dr Erasmus Darwin (1731–1802) of Lichfield and placental respiration. Arch. Dis. Child. Fetal Neonatal Ed..

[8] Hutton EK, Hassan ES (2007). Late vs early clamping of the umbilical cord in full-term neonates: systematic review and meta-analysis of controlled trials. JAMA.

[9] Rabe, H., Reynolds, G. J. & Diaz-Rosello, J. L. Early versus delayed umbilical cord clamping in preterm infants. in *Cochrane Database of Systematic Reviews* (ed. Rabe, H.) CD003248 (John Wiley & Sons, Ltd, 2004). 10.1002/14651858.CD003248.pub2 10.1002/14651858.CD003248.pub2 15495045

[10] Fogarty M (2018). Delayed vs early umbilical cord clamping for preterm infants: a systematic review and meta-analysis. Am. J. Obstet. Gynecol..

[11] Rabe, H., Diaz-Rossello, J. L., Duley, L. & Dowswell, T. Effect of timing of umbilical cord clamping and other strategies to influence placental transfusion at preterm birth on maternal and infant outcomes. *Cochrane database Syst. Rev*. CD003248, 10.1002/14651858.CD003248.pub3 (2012). 10.1002/14651858.CD003248.pub3 22895933

[12] National Institute for Health and Care Excellence. Intrapartum care for healthy women and babies (2014). 32212591

[13] WHO recommendations Intrapartum care for a positive childbirth experience. *World Heal. Organ*. (2018). 30070803

[14] American College of Nurse Midwives. Delayed Umbilical Cord Clamping.

[15] Committee on Obstetric Practice (2017). Committee Opinion No. 684: Delayed Umbilical Cord Clamping After Birth. Obstet. Gynecol..

[16] Al-Wassia H, Shah PS (2015). Efficacy and safety of umbilical cord milking at birth: a systematic review and meta-analysis. JAMA Pediatr..

[17] Katheria AC (2018). Umbilical Cord Milking: A Review. Front. Pediatr..

[18] Katheria A (2019). Association of Umbilical Cord Milking vs Delayed Umbilical Cord Clamping With Death or Severe Intraventricular Hemorrhage Among Preterm Infants. JAMA.

[19] Jaiswal P, Upadhyay A, Gothwal S, Chaudhary H, Tandon A (2015). Comparison of Umbilical Cord Milking and Delayed Cord Clamping on Cerebral Blood Flow in Term Neonates. Indian J. Pediatr..

[20] Jaiswal P (2015). Comparison of two types of intervention to enhance placental redistribution in term infants: randomized control trial. Eur. J. Pediatr..

[21] Wu H, Chen X, Wang P, Wang Q (2017). Effects of placental cord drainage in the third stage of labour: A meta-analysis. Sci. Rep..

[22] Katheria AC, Truong G, Cousins L, Oshiro B, Finer NN (2015). Umbilical Cord Milking Versus Delayed Cord Clamping in Preterm Infants. Pediatrics.

[23] Safarulla A (2017). A review of benefits of cord milking over delayed cord clamping in the preterm infant and future directions of research. J. Matern. Fetal. Neonatal Med..

[24] Polona Mivšek, A. Umbilical Cord Management and Stump Care in Normal Childbirth in Slovenian and Croatian Maternity Hospitals. *Acta Clin. Croat*., 10.20471/acc.2017.56.04.27 (2017). 10.20471/acc.2017.56.04.27 29590735

[25] Boere I (2015). Current practice of cord clamping in the Netherlands: a questionnaire study. Neonatology.

[26] Leslie MS, Greene J, Schulkin J, Jelin AC (2018). Umbilical cord clamping practices of U.S. obstetricians. J. Neonatal. Perinatal. Med..

[27] Ibrahim NO (2017). Current umbilical cord clamping practices and attitudes of obstetricians and midwives toward delayed cord clamping in Saudi Arabia. Ann. Saudi Med..

[28] María de la Fe Rodríguez-Muñoz, Laura Vallejo Slocker, María Eugenia Olivares Crespo, Nuria Izquierdo Méndez, C. S. y H.-N. Le. *Propiedades psicométricas del postpartumdepressionpredictorsinventory- revised- versión prenatal en una muestra española de mujeres embarazadas*. *Revista Española de Salud Pública* **91**, (Ministerio de Sanidad y Consumo, 2017). PMC11587386 29231186

[29] Ortigosa Gómez S (2015). Concentraciones plasmáticas de 25-OH vitamina D y parathormona en sangre de cordón umbilical. Rev. Esp. Salud Publica.

[30] Ortiz-Esquinas I (2019). Variability of Clinical Practice in the Third Stage of Labour in Spain. J. Clin. Med..

[31] Instituto Nacional de Estadística Estadística de profesionales sanitarios colegiados (2017).

[32] Evolución anual del número de obstetras y ginecólogos en activo en el sector sanitario en España de 2000 a 2016. *Estatista* (2018).

[33] Del Niño, S. & Adolescente, D. *Más allá de la supervivencia: Prácticas integrales durante la atención del parto, beneficiosas para la nutrición y la salud de madres y niños*.

[34] Katheria AC, Lakshminrusimha S, Rabe H, McAdams R, Mercer JS (2017). Placental transfusion: a review. J. Perinatol..

[35] National Institute for Health and Care. *Intrapartum Care: Care of Healthy Women and Their Babies During Childbirth*. (RCOG Press, 2007). 21250397

[36] WHO. Delayed Clamping *of the Umbilical Cord to Reduce Infant Anaemia*.

[37] Hutchon DJR (2015). Ventilation before Umbilical Cord Clamping Improves Physiological Transition at Birth or ‘Umbilical Cord Clamping before Ventilation is Established Destabilizes Physiological Transition at Birth’. Front. Pediatr..

[38] Farrar D, Tuffnell D, Airey R, Duley L (2010). Care during the third stage of labour: A postal survey of UK midwives and obstetricians. BMC Pregnancy Childbirth.

[39] Royal College of Obstetricians and Gynaecologists. *Clamping of the Umbilical Cord and Placental Transfusion* (2015).

[40] Dalheim A, Harthug S, Nilsen RM, Nortvedt MW (2012). Factors influencing the development of evidence-based practice among nurses: a self-report survey. BMC Health Serv. Res..

[41] March MI, Hacker MR, Parson AW, Modest AM, de Veciana M (2013). The effects of umbilical cord milking in extremely preterm infants: a randomized controlled trial. J. Perinatol..

[42] Perrone B, Ghirardello S, “Italian Survey on Placental Transfusion Group” (2017). Placental Transfusion Strategies in Italy: A Nationwide Survey of Tertiary-Care Delivery Wards. Am. J. Perinatol..

[43] Hosono S (2009). Blood pressure and urine output during the first 120 h of life in infants born at less than 29 weeks’ gestation related to umbilical cord milking. Arch. Dis. Child. Fetal Neonatal Ed..

[44] Krueger MS (2015). Delayed cord clamping with and without cord stripping: a prospective randomized trial of preterm neonates. Am. J. Obstet. Gynecol..

[45] Rabe H, Sawyer A, Amess P, Ayers S, Brighton Perinatal Study Group (2016). Neurodevelopmental Outcomes at 2 and 3.5 Years for Very Preterm Babies Enrolled in a Randomized Trial of Milking the Umbilical Cord versus Delayed Cord Clamping. Neonatology.

